# Sarcopenia in Women with Anorectal Dysfunctions—A Female Sarcopelvic Study

**DOI:** 10.3390/jcm13237273

**Published:** 2024-11-29

**Authors:** Ana Margarida Duarte da Silva Vieira, Sandra Pais, Viviana Martins, Barbara Castelo, Miguel Mascarenhas Saraiva

**Affiliations:** 1 Unidade Local de Saúde do Algarve, Unidade de Portimão de Gastrenterologia, 8500-338 Portimão, Portugal; viviana.martins@chua.min-saude.pt (V.M.); barbara.castelo@ulsalg.min-saude.pt (B.C.); 2 Faculdade de Medicina e Ciências Biomédicas, Universidade do Algarve, Campus de Gambelas, 8005-139 Faro, Portugal; 3 Comprehensive Health Research Center, Universidade de Évora, 7004-516 Évora, Portugal; spais@uevora.pt; 4 ManoPH, 4000-432 Porto, Portugal; miguelms.manoph@gmail.com; 5 Hospital CUF, 4100-180 Porto, Portugal

**Keywords:** pelvic floor, fecal incontinence, defecation, sarcopenia

## Abstract

**Background:** Anorectal dysfunctions (ARDs) include fecal incontinence (FI) and functional defecation disorders (FDDs). The pelvic floor muscles play a central role in the physiology of continence and defecation. We aimed to investigate the prevalence of sarcopenia in a female group with anorectal dysfunctions and compare them with a healthy female age-matched group. As secondary objectives, the relationship between anorectal dysfunction outcomes and sarcopenia was analyzed. **Methods:** We conducted a single-center cross-sectional, interventional, controlled, and double-blind study involving female adults admitted to an ARD outpatient clinic assessed for FI and/or FDD. A control group was also included of age-matched women without ARD. Sarcopenia was evaluated in the entire cohort, according to the latest criteria. Statistical analysis was performed using SPSS software v.29, considering a confidence interval of 95%. **Results:** A total of 130 participants were included, equally divided by the two groups. The median age was 64 years. Both groups were also similar regarding body mass index (BMI), physical activity index values, and dietary patterns. Among the 130 investigated women, there were no cases of confirmed sarcopenia or severe sarcopenia, but 15 women (11.5%) had probable sarcopenia or dynapenia. The case group had significantly more probable sarcopenia than women in the control group (14 (21.5%) vs. 1 (1.5%), *p* < 0.001). The presence of relevant comorbidities, such as irritable bowel syndrome (IBS), urinary incontinence (UI), and meat dietary pattern (MDP), was a risk factor for probable sarcopenia. The binomial logistic regression analysis showed that probable sarcopenia (OR 3.9; CI 1.1–14.1, *p* = 0.039) was associated with a worse treatment response. **Conclusions:** Probable sarcopenia or dynapenia was significantly more prevalent in women with ARD and was a predictive factor of a worse treatment response, regardless of the ARD severity. Concomitant UI, MDP, IBS, and psychiatric conditions were significantly associated with dynapenia. The inclusion of the evaluation of sarcopenia in these patients should be considered.

## 1. Introduction

IF and FDD are functional gastrointestinal diseases, currently recognized as brain–gut axis disorders [1,2].

The pelvic floor structures include muscular tissues of the pelvic floor and their neural connections, and the fascial (connective tissue) layers surrounding the pelvic floor muscle fibers/fascicles composing and working like a neuro-mio-fascial functional unit [3]. These muscles, at rest, are tonically and simultaneously contracted, in order to provide pelvic floor tone, support the pelvic organs, and maintain continence [4]. The physiology of continence and defecation requires coordination of pelvic floor and anal sphincter muscles, intact somatic, parasympathetic, and enteric nervous systems, and voluntary and involuntary mechanisms [5,6].

Sarcopenia is a progressive and generalized skeletal muscle disorder, with specific diagnostic criteria [7]. Sarcopenia is defined as a simultaneous loss of muscle function and mass or quality [7,8]. Sarcopenia used to be recognized as an aging-related disease, affecting up to 40% of the elderly population, but currently, it is known that sarcopenia often appears earlier in life [7,8,9].

At the moment, we do not have prevalence data of sarcopenia specifically in patients with ARD, regardless of age.

Therefore, we assumed that women with ARD could have a higher sarcopenia prevalence than women of similar age without ARD, independently of their age, and sarcopenia could negatively affect the outcome of these patients.

In this study, we aimed to investigate the prevalence of sarcopenia in a female group with ARD and compare it with a healthy female age-matched group to examine the relationship between ARD severity and sarcopenia.

## 2. Material and Methods

### 2.1. Participants and Study Setting

We conducted a single-center cross-sectional, interventional, controlled, and double-blind study involving community female adults admitted to the ARD outpatient clinic at The Algarve University Hospital and assessed for FI and/or FDD, between September 2017 and September 2023. Establishing the diagnosis and assessing the severity of the disease before and after the first-line therapeutic approach, which is based on bowel movement regularization with normal stool consistency and pelvic floor rehabilitation, are regular procedures of our clinical assessment, which are recorded in the patient’s medical registry.

In the diagnostic assessment, the Rome IV criteria were used for FI, while either the Rome IV or RAO criteria were applied for FDD. All patients underwent laboratory evaluations, including thyroid function tests for all, celiac disease serology, and fecal calprotectin tests for those with increased stool frequency and/or decreased stool consistency. Parathyroid hormone levels were also assessed in patients with FDD. Women over the age of 45 underwent a total colonoscopy. A proctological exam with anoscopy was performed on all patients.

Pelvic floor rehabilitation was performed in 83% of patients (54 out of 65). During the study period, no patients underwent surgery.

An age-matched control group was also recruited from the endoscopic department, other appointments of our department, or patient companions that did not have FI or FDD.

Exclusion criteria, for both groups, were refusal of consent to participate, missing data and/or follow-up, oncologic disease diagnosed up to 5 years and/or under surgical and/or systemic treatment or end-of-life care stage, renal replacement treatment or chronic kidney disease pre-dialysis, a chronic obstructive pulmonary disease with oxygen therapy, acute or chronic immobility, severe malnutrition (Global Leadership Initiative on Malnutrition (GLIM) criteria) [10], and all patients with any condition affecting physical capacity independently of sarcopenia grade (e.g., dementia, neurologic, neuromuscular, or orthopedic conditions, and psychiatric diseases).

All women underwent sarcopenia evaluation. The muscle function and mass were assessed by a trained nutritionist who was blinded to all other clinical data of the participants.

### 2.2. Data Collection

Information on patient demographic characteristics was recorded, as well as smoking habits, physical exercise using the International Physical Activity Questionnaire—Short Form (IPAQ-SF) [11], anthropometric data (weight, height, and BMI), dietary patterns (meat dietary pattern (MDP) or fruit–vegetable dietary pattern (FVDP)) [12], relevant comorbidities for bowel function like diabetes, thyroid disorders, neurologic or psychiatric conditions, scleroderma, fibromyalgia and dermatomyositis, medication history, and obstetric and gynecologic data; assessment of ARD–FI and FDD were evaluated using the Wexner Scale [13] and Renzi Obstructed Defecation Syndrome (ODS) scores [14], respectively, before and after treatment. For the evaluation of treatment response, it was also gauged the number of involuntary anal losses and number of defecatory facilitations. All questionaries used have been translated into Portuguese and validated, and these were the versions used.

Sarcopenia was screened and diagnosed, according to the consensus of the European Working Group on Sarcopenia in Older People (EWGSOP) [7], namely the recommended tests to measure muscle strength, muscle mass, and physical performance.

### 2.3. Sarcopenia Definition

Based on the EWGSOP criteria [7], sarcopenia was diagnosed when both low skeletal muscle mass index scores by bioelectrical impedance analysis (BIA) and low muscle strength by handgrip strength (HGS) were present.

The EWGSOP2 algorithm was followed. The first step was the application of the SARC-F questionnaire, the Portuguese version [15], to find cases. If SARC-F was equal to or higher than 4 [16], muscle strength was assessed using the Saehan Squeeze dynamometer SH5008 (serial number 004489), and values lower than 16 kg defined probable sarcopenia [7]. HGS was measured in a sitting position, with the elbow in 90° flexion and the wrist in a neutral position [17]. Participants were asked to apply maximum grip strength three times with their dominant hand. The maximal measured grip strength was regarded as the HGS [17].

A definitive diagnosis of sarcopenia also required a skeletal muscle mass index score via BIA lower than 5.5 kg/m^2^ [7]. The instrument used was a multifrequency segmental body composition monitor, Tanita MC-780MA P, a validated BIA device that measures the sarcopenia index.

Whenever sarcopenia was diagnosed, the Short Physical Performance Battery (SPPB) was implemented, and severe sarcopenia was considered when SPPB performance scores were 8 or less [18].

### 2.4. Definition of ARD

According to Rome IV [1], IF and FDD are included in ARD.

Recurrent uncontrolled passage of fecal material in an individual with a developmental age of at least 4 years, for the last 3 months, defines FI [1]. The Wexner Index [13] was used for severity evaluation.

The patient with FDD must satisfy diagnostic criteria for functional constipation and/or IBS with constipation and, during repeated attempts to defecate, there must be features of impaired evacuation, demonstrated by 2 of the 3 tests (abnormal balloon expulsion test, abnormal anorectal evacuation pattern with manometry or anal surface electromyography, or impaired rectal evacuation by imaging) [1]. These conditions need to be present during the last 3 months with symptom onset at least 6 months prior to diagnosis [1].

Rao, in 2020, updated these criteria and defined dyssynergic defecation requiring the presence of 3 conditions (symptoms of chronic constipation based on Rome IV criteria, a pattern of dyssynergic defecation, and, at least 1 other quantifiable measure of abnormal defecation, like an abnormal balloon expulsion test, a prolonged delay in colonic transit, or an incomplete evacuation during defecography) [19]. Rao defined the dyssynergic pattern of defecation as a paradoxical anal contraction (an increase in anal sphincter pressure), inadequate relaxation of the resting anal sphincter pressure (<20%), or inadequate effort of abdominorectal muscles on anorectal manometry, imaging, or electromyographic recordings [19].

Since our center does not have anorectal manometry, patients who met the criteria of Rome IV or Rao were included.

The ODS score [14] was used for the evaluation of FDD severity [20].

### 2.5. Definition of Severe Malnutrition

The GLIM was convened by several of the major global clinical nutrition societies. In 2018, the GLIM recommended, for the diagnosis of malnutrition, the combination of at least one phenotypical criterion (weight loss, low BMI, or reduced muscle mass) and one etiological criterion (reduced food intake or absorption, increased disease burden, or inflammation) [10].

Stage 2 or severe malnutrition is established if the patient meets GLIM criteria and presents a weight loss higher than 10% within the past 6 months or >20% beyond 6 months, or presents with a BMI < 18.5 kg/m^2^ if their age is <70 yr or BMI is <20 kg/m^2^ if their age is ≥70 yr [10].

### 2.6. Outcome Measures

For the main goal, the endpoint was the comparison between the proportion of women with sarcopenia and FI and/or FDD and the proportion of sarcopenia in the control group without FI and/or FDD.

The secondary endpoints were the correlation of sarcopenia and ARD treatment response, FI and FDD severity, the disorder type, the presence of other compartment disorders, and the relation with age, BMI, dietary patterns, smoking habits, physical exercise, comorbidities, or medical history.

### 2.7. Ethics

This study was conducted in accordance with the International Conference on Harmonization guidelines, respecting the 1964 Declaration of Helsinki and Good Clinical Practice. This study was approved by our institutional health ethics committee (141/UAIP/2022). Written informed consent was obtained from all participants.

### 2.8. Sample and Statistical Analysis

The sample was composed of all patients or participants fulfilling the selection criteria and with patients who gave informed consent. The control group was of equal number and similar age.

All analyses were performed using SPSS software (version 29). The results are reported as median and interquartile range for numerical variables and number (percentage) for categorical data.

The two groups were compared using the Mann–Whitney U test for continuous variables, verifying the nonadherence of the data to a normal distribution, and Pearson’s Chi-square test and Fisher’s exact test for categorical variables.

To verify the difference between the case group and the control group of sarcopenia prevalence, the latter was classified as “no” (SARC F < 4) and the former as “yes” (probable sarcopenia (SARC F ≥ 4 and HGS < 16 kg), confirmed or severe sarcopenia), using a Chi-square test.

For the correlation analysis, a Chi-square test and binomial logistic regression estimating association measures—Odds Ratios (ORs) and respective confidence intervals of 95%—were used.

The results were considered statistically significant for *p*-values less than 0.05.

### 2.9. Results

A total of 130 participants were included, divided into the case group (n = 65) and the control group (n = 65). The baseline of the comparable characteristics of the enrolled participants are summarized in Table 1. According to the study design, there was agreement between the ages of the two groups, with a median age of 64 years. Both groups were also similar regarding BMI, physical activity index values, and dietary patterns. The median BMI was 25.6 kg/m^2^ (IQR 22.2–28.6 kg/m^2^). Only 13% of the participants presented health-enhancing physical activity (HEPA activity) and 60% fulfilled a FVDP.

The median follow-up time of the case group was 37 months (IQR 17.25–59.25 months); FI was the reason for follow-up in 44 (67.7%) women, and FDD in 38 (58.5%) women. Both conditions were simultaneously present in 17 (26.2%) women. Smoking history was identified in 12 (18.5%) cases with a mean tobacco load of 18.1 smoking pack-years. Other relevant comorbidities were found in 32 (49.2%) women, and 38 (58.5%) patients were using one or more medications, which were potentially causing the women’s functional symptoms. The antidepressant and benzodiazepine drugs were the most implicated drugs, in 29 (44.6%) and 25 (38.5%) cases, respectively. As expected, psychiatric conditions were the most present disorder, in 25 (38.5%) patients, followed by diabetes in 10 (15.4%), thyroid problems in 5 (7.7%), fibromyalgia in 4 (6.1%) and scleroderma in 1 (1.5%). According to the Rome IV criteria [1], 19 (29.2%) patients had a diagnosis of IBS. The mixed type was the most frequent (9 patients, 47.4%), followed by the constipation type (6 cases, 31.6%) and diarrhea type (4 patients, 21.1%). There was no association between IBS and FI or FDD.

Only 4 (6.2%) women were nulliparous, and 42 (64.6%) women had a history of two or more childbirths. A dystocic delivery history and a high weight of the newborn (considered weight higher than 3500 g) were documented in 18 (27.7%) cases and in 35 (53.8%) cases, respectively. There was no association between FI or FDD and a dystocic delivery history (*p* = 0.558 and *p* = 0.785, respectively) or a high newborn weight (*p* = 1.000 and *p* = 0.614, respectively).

In the case group, 47 (72.3%) patients were post-menopausal women with a median of 252 months (IQR 168–324 months) of menopause time.

**Figure 1:**
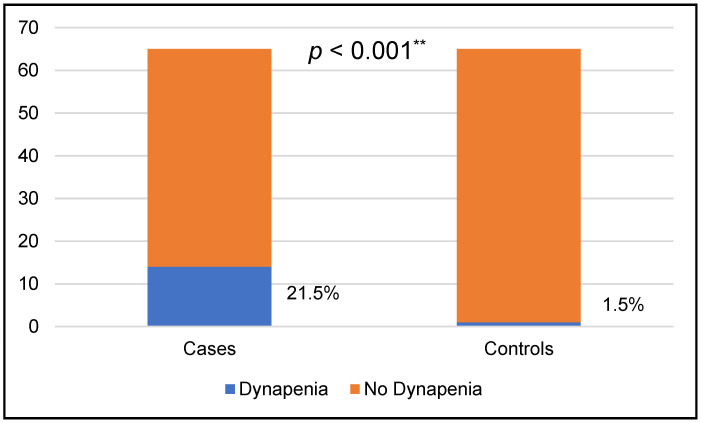
Prevalence of probable sarcopenia or dynapenia. ** ꭕ^2^ test.

Among the 130 women investigated, 15 (11.5%) had probable sarcopenia, and 115 (88.5%) had no sarcopenia. There were no cases of definitive or severe sarcopenia. The prevalence of probable sarcopenia in women with FI and/or FDD was 21.5%. The case group had significantly more probable sarcopenia than women of the control group (14 (21.5%) vs. 1 (1.5%), *p* < 0.001) (Figure 1).

The only case of probable sarcopenia in the control group was a 43 y female, with a normal BMI (21.4 kg/m^2^), who was sedentary, following an FVDP, and who scored 4 on the SARC-F questionnaire and presented 12.2 kg of maximal HGS.

Sarcopenia screening was positive in a total of 20 individuals (15.4%), with 19 from the study group (29%) and 1 from the control group (1.5%). The median maximal HGS of these cases with a positive SARC-F was 11 kg (IQR 9.3–16.2 kg). None of these individuals had low muscle mass via BIA, in which the median value was 7.5 kg/m^2^ (IQR 6.9–7.9 kg/m^2^).

Probable sarcopenia was not associated with ARD type (FI or FDD), FI type, smoking habits, overweight and/or obesity, medication, or IPAQ-SF. When stratifying age into two groups with cut-off points at 40 y, 60 y, and 80 y, we did not find any association between age and a decreased HGS (probable sarcopenia) (Table 2).

However, the patients with ARD (IF and/or FDD) and UI have significantly more probability of probable sarcopenia (34.4% vs. 9.1%, *p* = 0.014), with a V Cramer factor of 0.31 and an OR of 5.2 (CI 1.3–21.1) (Table 2).

The MDP was significantly associated with probable sarcopenia (44% vs. 11%, *p* < 0.001), with a V Cramer factor of 0.43 and an OR of 9.7 (CI 2.3–40) (Table 2).

The presence of relevant comorbidities increased the risk of probable sarcopenia (a V Cramer factor of 0.31 and an OR of 5.2 (CI 1.3–21.1), and only 9.1% of the patients without associated diseases presented probable sarcopenia compared to 34.4% of the patients with relevant comorbidities (*p* = 0.014). The simultaneous presence of IBS was associated with a higher risk of probable sarcopenia (42.1% vs. 13%, *p* = 0.014, V Cramer factor 0.32, OR 4.85 (CI 1.4–16.9)) (Table 2).

Using Wexner and ODS scores, probable sarcopenia was not associated with a higher severity of ARD. However, probable sarcopenia was significantly associated with a worse treatment response (71.4% vs. 39.2%, *p* = 0.033, V Cramer factor 0.27, OR 3.87 (CI 1.1–14.1)) (Table 2).

The binomial logistic regression analysis showed that sarcopenia (OR 3.9; CI 1.1–14.1, *p* = 0.039) but not a more severe disease was associated with a worse treatment response (Table 3). Other factors associated with probable sarcopenia like the dietary pattern, the presence of UI, IBS, or relevant comorbidities were not predictive of treatment response.

## 3. Discussion

Our population is notable for having a considerable percentage of young women; 38.5% of the women are under 60 years old (35.4% in the study group, 41.5% in the control group). Furthermore, the two groups are similar not only in terms of age, but also in BMI, dietary patterns, and level of physical exercise. Obesity is present in only 26 individuals (20%). It is also important to highlight the exclusion criteria, which exclude from the study women with a low BMI and/or any clinical condition that could cause malnutrition and/or sarcopenia.

The authors consider that, in addition to the statistically significant difference in the percentage of probable sarcopenia between the two groups (14 (21.5%) vs. 1 (1.5%), *p* < 0.001), the percentage of more than 21% is quite significant and puts this group of patients at risk of probable sarcopenia. In fact, the most recent meta-analysis published on global sarcopenia prevalence shows that for the population aged 60 y or more, in Europe, using the same diagnostic criteria, the percentage of sarcopenia was 1%, highlighting the absence of data for younger populations of sarcopenia and dynapenia prevalence [9].

Our results support the association between FI and/or FDD and an increased risk of sarcopenia and reduced muscle strength, known as dynapenia. Soytas et al. found a positive correlation between HGS and pelvic floor muscle strength of 92 women with the complaint of urinary incontinence and concluded that a low HGS could be a marker of pelvic floor muscle weakness [21]. Suskind et al., in a study conducted on older women, showed that the decline in muscle strength over time was associated with new and persistent symptoms of stress urinary incontinence [22].

The study of Erdogan et al., which evaluated the relationship between UI and sarcopenia in older women, independently of nutrition status and using less restrictive cut-offs for muscle function and mass, verified an association to sarcopenia adjusted by weight, but not to a low HGS [23].

There are only two published studies with data on FI: one prevalence study of sarcopenia in older women with pelvic floor dysfunction, but with the inclusion of only nine cases of FI, failing to prove its association with sarcopenia [24]; and a second study with dwelling elderly patients of both gender, with dysphagia and 74% malnutrition, found that sarcopenia (according to the criteria of the Asian Working Group for Sarcopenia) was an independent risk factor for FI [25].

To our knowledge, this is the first controlled study to evaluate the prevalence of sarcopenia in women of all ages with ARD, using the new diagnostic criteria of sarcopenia and with strict exclusion of malnutrition.

According to the revised consensus of sarcopenia, muscle strength became a forefront criterion for sarcopenia diagnosis because it is predictive of adverse outcomes [7]. This condition was documented as significantly more prevalent in our group of patients. In none of these cases, definitive sarcopenia was not confirmed, which is due, on one hand, to the use of BIA as a diagnostic method without proven correlation with the gold standard method Dual X-ray Absorptiometry (DEXA) [26], lacking validated cut-offs for different populations [27], and not allowing for the assessment of muscle quality. Assessing muscle quality is also a diagnostic criterion for definitive sarcopenia and it seems to be correlated with muscle function [28]. Nevertheless, we emphasize the median muscle mass value of these women, 7.5 kg/m^2^, which is less than 2 kg/m^2^ above the diagnostic cut-off.

The FVDP was the factor with the greatest protective effect against the development of dynapenia. This result had already been suggested in the systematic review published in 2018, although with weak evidence, particularly regarding its association with muscle strength [29]. Perälä et al. demonstrated that women with higher intakes of fruits and vegetables had higher HGS [30]. More recently, a meta-analysis found that protein consumption reduces the risk of sarcopenia, particularly when the protein is from plant sources [31].

The risk of sarcopenia was equally influenced by the presence of other comorbidities, particularly the most prevalent ones, such as psychiatric conditions, which are known to be associated with a higher risk of sarcopenia [32] and dynapenia [33,34,35], when discussing a bidirectional cause–effect relationship.

The association with IBS was not justified by dietary patterns, the presence of comorbidities, age, a greater severity of ARD, or a worse response to treatment, as no association was documented between these factors. Like ARD, being a disease of the brain–gut axis, they likely share similar pathophysiological mechanisms and could be important to study sarcopenia in these patients.

We also documented an association with UI, suggesting that the presence of disease in various compartments of the pelvic floor may be related to greater muscle weakness.

Surprisingly, greater disease severity was not demonstrated in women with probable sarcopenia, which raises questions about the effect of dynapenia on the pathophysiology of ARD. However, further studies are needed, specifically those assessing markers of less strength and increased laxity of pelvic floor muscles in more homogeneous groups, exclusively with FI or FDD.

Finally, patients with dynapenia, despite not having a greater disease severity, showed a worse treatment response. This is in accordance with the results of Kido Y et al., which revealed that in post-stroke patients, sarcopenia negatively affects the recovery of urinary and fecal independence [36]. Furthermore, the improvement of sarcopenia and muscle strength is associated with better sphincter control [37].

This study has some limitations; particularly, its cross-sectional nature limits the ability to draw causal conclusions. In addition, it was conducted in a single region of the country and the sample size may affect the generalizability of the results.

## 4. Conclusions

The studied population with FI and/or FDD has significantly more probable sarcopenia than the population of women without ARD, as demonstrated by the loss of HGS. Patients with dynapenia are more difficult to treat, as dynapenia is independently associated with a worse treatment response. Concomitant UI, MDP, IBS, and psychiatric disorders were risk factors for dynapenia.

The authors recommend the assessment of sarcopenia in patients with ARD and further research in this area to identify, prevent, and treat these patients.

## Figures and Tables

**Table 1:** Comparison of baseline characteristics of participants.

	Case Group (n = 65)	Control Group (n = 65)	Total (n = 130)	*p*-Value
Age, y	66 (48–74.5)	62 (50.5–72.5)	64 (48–73.2)	0.537 *
Body mass index, kg/m^2^	25.2 (22.2–29.1)	25.8 (22.2–27.9)	25.6 (22.2–28.6)	0.955 *
IPAQ-SF				0.417 **
Inactive	33 (50.8)	35 (53.8)	68 (52.3)	
Minimally active	21 (32.3)	24 (36.9)	45 (35)	
HEPA active	11 (16.9)	6 (9.2)	17 (13)	
Dietary patterns				0.720 **
FVDP	40 (61.5)	38 (58.5)	78 (60)	
MDP	25 (38.5)	27 (41.5)	52 (40)	

Numeric variables are presented as median (interquartile range); categorical variables are presented as absolute number (%). * Mann–Whitney U test; ** χ^2^ test.

**Table 2:** Clinical data of the case group between the presence or absence of probable sarcopenia.

	Total (n = 65)	Sarcopenia+ (n = 14)	Sarcopenia− (n = 51)	*p*-Value
FI (y/n) ^ƚ^	65	10 (22.7)/4 (19)	34 (77.3)/17 (81)	0.503 **
FDD (y/n) ^ƚ^	65	7 (18.4)/7 (25.9)	31 (81.6)/20 (74.1)	0.335 **
Smoking habits (y/n) ^ƚ^	65	2 (16.7)/12 (22.6%)	10 (83.3)/41 (77.4)	0.494 **
Overweight or obesity (y/n) ^ƚ^	65	8 (24.2)/6 (18.8)	25 (75.8)/26 (81.3)	0.407 **
Medication (y/n) ^ƚ^	65	10 (26.3)/4 (14.8)	28 (73.7)/23 (85.2)	0.212 **
Age				
<80 y/≥80 y	65	11 (19.3)/3 (37.5)	46 (80.7)/5 (62.5)	0.228 **
<60 y/≥60 y	65	2 (8.7)/12 (28.6)	21 (91.3)/30 (71.4)	0.056 **
<40 y/≥40 y	65	0 (0)/14 (24.6)	8 (100)/43 (75.4)	0.126 **
IPAQ-SF				0.206 *
Inactive	33	10 (30.3)	23 (69.7)	
Minimally active	21	3 (14.3)	18 (85.7)	
HEPA active	11	1 (9.1)	10 (90.9)	
Dietary pattern				*<0.001* **
Meat dietary pattern	35	11 (44)	14 (56)	
Fruit–vegetable pattern	30	3 (7.5)	37 (92.5)	
+UI (y/n) ^ƚ^	65	11 (34.4)/3 (9.1)	21 (65.6)/30 (90.9)	*0.014* **
Comorbidities (y/n) ^ƚ^	65	11 (34.4)/3 (9.1)	21 (65.6)/30 (90.9)	*0.014* **
IBS (y/n) ^ƚ^	65	8 (42.1)/6 (13)	11 (57.9)/40 (87)	*0.014* **
Treatment response (y/n)	65	4 (11.4)/10 (33.3)	31 (88.6)/20 (66.7)	*0.033* **

FI, fecal incontinence; FDD, functional defecation disorders; UI, urinary incontinence. Categorical variables are presented as absolute number (%). ƚ y, yes; n, no; ** Fisher’s exact test; * Pearson’s Chi-square test.

**Table 3:** Binomial logistic regression analysis with treatment response as dependent variable.

	B	*p*-Value	OR	CI
Model 1				1.2–17.5
Probable sarcopenia	1.535	0.024	4.643	0.1–1.7
Severe disease	−0.801	0.246	0.449	
Constant	0.182	0.763	1.2	
Model 2				1.1–14.1
Probable sarcopenia	1.355	0.039	3.875	
Constant	−0.438	0.127	0.645	

## Data Availability

The original contributions presented in the study are included in the article, further inquiries can be directed to the corresponding author/s.
